# Internet addiction mediates the association between cyber victimization and psychological and physical symptoms:moderation by physical exercise

**DOI:** 10.1186/s12888-020-02548-6

**Published:** 2020-04-03

**Authors:** Ling Lin, Jianbo Liu, Xiaolan Cao, Siying Wen, Jianchang Xu, Zhenpeng Xue, Jianping Lu

**Affiliations:** Department of Child Psychiatry of Shenzhen Kangning Hospital, Shenzhen Mental Health Center, School of mental health, Shenzhen University, Shenzhen, 518003 China

**Keywords:** Cyberbullying, Cyber victimization, Exercise, Internet addiction, Psychological and physical symptoms, Physical activity

## Abstract

**Background:**

The potential mechanisms underlying cyber victimization and the resulting psychological and physical symptoms remain unclear. Thus, the present study investigated whether Internet addiction mediates the association between peer victimization (e.g., cyberbullying) and psychological and physical symptoms. Furthermore, it was assessed whether physical exercise moderates the hypothetical mediation.

**Methods:**

1854 students from 11 middle and high schools in Shenzhen, Guangdong Province, China, were sampled for this study. Psychological and physical symptoms were assessed using the World Health Organization Quality of Life-BREF, while Internet addiction was evaluated using the Internet addiction test by Young. Cyber victimization was measured using a single question. In addition, this study examined whether Internet addiction mediated the association between cyber victimization and both psychological and physical symptoms. Additional work was conducted to test if physical exercise played a moderating role in the mediation hypothesized above. Mediation and moderation were analyzed using PROCESS macro for SPSS.

**Results:**

Regression analysis showed that both cyber victimization (β = − 0.102, *p <* 0.05) and Internet addiction (β = − 0.278, *p <* 0.05) significantly predicted psychological and physical symptoms and demographic variables were controlled. Further mediation analysis suggested that Internet addiction mediated the relationship between cyber victimization and psychological and physical symptoms. The 95% CI (confidence interval) of the direct effect was (− 4.283, − 1.696) and the indirect effect (− 1.904, − 0.820), respectively, excluding zero. Finally, moderation analysis indicated that physical exercise moderated the relationship between Internet addiction and psychological and physical symptoms (*p* = 0.047).

**Conclusions:**

Internet addiction plays a mediating role in the association between cyber victimization and both psychological and physical symptoms, Thus, addressing Internet addiction among cyberbullying victims is worthwhile. Furthermore, physical exercise alleviates negative impacts on health and should thus be promoted.

## Background

Online victimization by peers as well as its adverse consequences have become a serious public health problem. According to a review published in 2015, most studies found that the proportion of students who reported to be victims of cyberbullying ranged from 20 to 40% [1]. Data from the United States in 2015 reported that 15.5% of high school students have been bullied online [2]. Similarly, about 15% of teenagers reported being victims of cyberbullying in countries such as France, England, and Spain [3–5]. Among the Chinese population, the prevalence of cyber victimization has been reported as 18.4 and 11.9% among adolescents from Taiwan and Hong Kong, respectively [6, 7]. However, a recent study, conducted in ShenZhen Guangdong, China, showed a lower prevalence (8.7%) of cyber victimization among middle-school students [8]. In addition, a relatively higher rate of cyber victimization has also been reported (37.3 and 32%) among teenage students in Romania and Bangladesh, respectively [9, 10].

To date, a number of studies have been conducted that examined the impact of peer victimization on their physical and mental health. It has been shown that peer victims report higher rates of suicidal ideation at the age of 13 and conducted suicide attempts at the age of 15 compared with those who have not been victimized [11]. Additionally, the long-term impacts of bullying by peers on mental health have been found to persist into young adulthood [12]. With respect to online peer victimization, increased internalizing disorders [3],externalizing disorders, substance-use problems [13, 14], and a high risk of depression have been identified in victims of cyberbullying compared with nonvictims [4, 6]. The victims of cyberbullying showed significantly higher rates of suffering from psychiatric disorders compared with non-victims [10] . A study conducted in Australia has consistently suggested poor mental health among youths who experienced cyber victimization [15]. Furthermore, experience of cyber victimization was found to be related to posttraumatic stress disorder (PTSD) symptoms via studying teenagers sent to the emergency department [16]. A meta-analysis demonstrated the link between suicides and both types of peer victimization (cyberbullying and face-to-face bullying), within which, cyberbullying exerted a stronger effect on suicidal ideation compared with face-to-face bullying [17]. With regard to gender differences, more emotional symptoms were observed in females, whereas more risk behavior was observed in males when subjected to cyber victimization [18]. In addition to psychological symptoms, somatic problems (such as headaches and abdominal pain) have been associated with cyberbullying [19]. Furthermore, cyberbullying victimization has been associate with a significant decrease in subjective wellbeing [20].

Given that a strong and reliable association exists between cyber victimization and critical physical and mental health problems, finding ways to intervene or prevent such problems is necessary. However, only few studies have explored the potential mechanisms underlying the links between cyber victimization and the negative outcomes mentioned above. One study conducted among Italian adolescents found that psychological resilience plays a mediating role between cyber victimization and emotional symptoms [21]. Family dinners were proposed to moderate the relationship between cyber victimization and mental health problems [13]. In addition, connectedness between students and school was identified to function as a moderator by alleviating the association between cyber victimization and suicide [22]. This study hypothesized that victims may search for other means, such as a virtual reality to cope with the pressures of being bullied. A Chinese study verified that peer victimization during 7th grade is a strong predictor for Internet-gaming addiction during the 9th grade [23]. Consistently, a significant association between peer victimization and pathological Internet use (assessed by the Young Diagnostic Questionnaire) has been reported in a study on German adolescents [24]. Moreover, Internet addiction was shown to be associated with cyber victimization [25, 26]. In addition, the bidirectional relationship between cyber victimization and Internet use was shown in a Chinese study, suggesting a higher likelihood of cyber victimization among youths with Internet risk behavior (e.g., chat with strangers, and post personal pictures) [6]. Internet use (more than two hours per day) was shown to predict a higher probability of being cyberbullied [9].

Internet addiction has also been considered as a psychological escape mechanism to avoid real-world problems [27] and has been proven to be associated with both mental and physical symptoms. Examples are the higher risk of Internet addition, inferior the mental health outcomes, suicidal ideation, depression, and anxiety [28, 29]. In addition, intense back pain, headaches, and increased body mass index have been observed among Internet-addicted youths [30]. Furthermore, Internet addiction has been identified to negatively impact the ophthalmologic system in the form of eye strain, and an increase of sleep disorders has been reported among Internet-addicted youths compared with non-addicted individuals [31]. Cyberbullying could occur via a special path that could lead to increased severe harm among individuals who have been bullied in their real life [32].

Based on previous research, this study hypothesized that cyber victimization is associated with Internet addiction, which in turn is related to both physical and mental health. Thus, the present study examined whether Internet addiction mediates the association between cyber victimization and both psychological and physical health.

In addition, problematic Internet use (PIU, measured through Internet addiction test), has been linked to decreased physical activity [33–35]. Physically active youths tend to obtain increased satisfaction from sleep and are less likely to develop Internet addiction compared with their peers who are physically inactive [36]. In fact, exercise rehabilitation has been applied as a measure to alleviate smartphone addiction [37].

Further studies supported that physical exercise can improve psychological and physical symptoms. Long-term physical exercise has been shown to improve negative symptoms in inpatients with mental health issues [38]. Moreover, regular exercise reduces anxiety and depression and improves self-esteem [39, 40]. From the perspective of neurobiology, physical activity leads to the release of endorphins, dopamine, noradrenaline, and serotonin, all of which can ease pain and promote a sense of euphoria and wellbeing [41]. Despite its positive effects, the role of physical exercise in the relationship between Internet addiction and both mental and physical health remains unknown. Hence, the second purpose of this study was to examine whether physical exercise plays a moderating role in the mediation posed above.

## Methods

### Participants

The participants of this study originated from 11 middle and high schools in Shenzhen, Guangdong Province, China, and were identified via stratified cluster sampling. A total of eight classes were chosen from each grade level. Ultimately, 48 classes in two districts were included in this study. All students from the selected classes were invited to complete questionnaires. Informed consent was obtained from participants before the study began. 2200 students were recruited, and a total of 1854 valid questionnaires were collected, indicating an efficiency of 84.3%. All participants and their parents or legal guardians provided verbal informed consent. The study protocol was approved by the ethics committees of the Shenzhen Kangning Hospital.

### Measurements

#### Physical exercise and cyber victimization

Physical exercise was evaluated by a single question: “How often do you exercise per week?” and was assessed using a 4-point scale (1 = none, 2 = 1–2 times per week, 3 = 3–5 times per week, and 4 = 5 times or more per week). Cyber victimization was measured with one question: “Have you ever been bullied on the Internet in the past year?” The value of cyber victimization was 0 when no victimization of cyberbullying was reported, and 1 when victimization of cyberbullying was reported.

#### Young’s internet addiction test

The Internet addiction test (IAT) is a scale developed by Young that screens whether users are addicted to the Internet as well as the severity of their addiction [42]. The scale consists of 20 items (e.g., Do you often make new friends online?), each of which rated on a 5-point Likert scale ranging from 1 (rarely) to 5 (always). Participants were considered addicted to the Internet when the total score of the calculated items was at least 40 points, where higher scores reflect a higher severity of Internet addition. The Chinese version of the IAT showed a satisfactory reliability and validity among Chinese students, with an internal consistency of 0.93 and a split-half reliability coefficient of 0.94 [43].

#### Short version of the World Health Organization quality of life-BREF

The World Health Organization Quality of Life-BREF (WHOQOL-BREF) [44], which is an abbreviated version of the WHOQOL-100 [45], was used to measure the quality of life. The WHOQOL-BREF consists of 26 items and assesses four domains of the quality of life, including physical health, psychological health, social relationships, and the environment. The former two domains (physical health and psychological health) were analyzed in the present work. Physical and psychological symptoms were obtained by adding scores of both selected domains. The higher these calculated scores, the better the health status. This measurement has been introduced in the Chinese population [46]. The WHOQOL-BREF showed satisfactory validity and reliability, with internal consistency (Cronbach’s α of 0.82) for the physical health domain and for the psychological health domain (Cronbach’s α of 0.81) [44].

### Statistical analysis

All analyses were conducted with SPSS version 24.0 (IBM, Armonk, NY), and descriptive statistics were employed to analyze demographic data. Independent sample T-tests were performed to examine the significance of variables (i.e., Internet addiction and psychological and physical symptoms) between the cyber-victimization group and non-cyber-victimization group. Subsequently, the Pearson correlation coefficients were calculated between Internet addiction and psychological and physical symptoms. A multiple linear regression analysis was conducted, controlling for demographic variables including age, gender, being an only child, academic performance, academic stress, physical exercise, and family income. The variables of Internet addiction and cyber victimization were regressed on the scores of psychological and physical symptoms.

In regard to the mediation model, both indirect and direct effects of cyber victimization via Internet addiction on psychological and physical symptoms were tested by PROCESS macro for SPSS version 24.0 using 2000 bootstrap samples. Cyber victimization was used as independent variable, Internet addiction was used as mediating variable, and psychological and physical symptoms were used as dependent variables. Similarly, physical exercise was the moderator in the moderation model, and the moderating effect of physical exercise on each path within the three model variables (i.e., cyber victimization, Internet addiction, and psychological and physical symptoms) was examined by PROCESS macro. The present study considered the effect significant at the 0.05 probability level if the resulting 95% confidence interval (CI) did not include zero.

## Results

### General demographic data

The distribution of the demographic information of participants, including age, gender, being an only child, and their academic performance is shown in Table 1. With respect to gender, almost half (49.3%) of the 1825 participants were male, and the mean ± standard deviation (M ± SD) of age was 15 ± 1.6 years (see Table 1).

**Table 1:** Demographic data

Data item	Description	*N* (%) / (*M ± SD*)
Age		15 ± 1.6 (years)
Gender	male	914 (49.3%)
	female	911 (49.1%)
	missing data	29 (1.6%)
Single child	yes	681 (36.7%)
	no	1058 (57.1%)
	missing data	115 (6.2%)
Academic performance	excellent	271 (14.6%)
	good	529 (28.5%)
	average	815 (44.0%)
	bad	208 (11.2%)
	missing data	31 (1.7%)
Academic stress	low	111 (6.0%)
	average	1098 (59.2%)
	high	638 (34.4%)
	missing data	7 (0.4%)
	missing data	10 (0.5%)
Physical exercise	yes	1670 (90.1%)
	no	180 (9.7%)
	missing data	4 (0.2%)
Internet addiction	yes	847 (45.7%)
	no	1007 (54.3%)
	missing data	0
Cyber victimization	yes	160 (8.6%)
	no	1694 (91.4%)
	missing data	0

*M:* Mean, *SD:* Standard deviation

### Independent sample T-test and correlation analysis

The independent sample t-test for cyber victimization on Internet addiction resulted in *T* = − 5.936, *p* < 0.001, which indicated that the differences in Internet addiction between the cyber victimization group and the non-cyber victimization group were statistically significant (see Table 2). Lower Internet addiction scores was observed when non-cyber victimization was reported. In contrast, higher Internet addiction scores was observed when cyber victimization was experienced. Similarly, significant differences were observed in the scores of psychological and physical symptoms between the cyber-victimization group and the non-cyber-victimization group, with *T* = 6.473, *p* < 0.001 (see Table 2). A higher score of psychological and physical symptoms indicates a relatively satisfactory overall health status when no cyber victimization occurs.

**Table 2:** Association between cyber victimization and Internet addiction as well as between cyber victimization and psychological and physical symptoms

		N	Mean ± SD	T	*p-*value
Internet addiction	Non-cyber victimization	1694	39.54 ± 13.98	−5.936	< 0.001
	cyber victimization	160	48.83 ± 19.33		
Psychological and physical symptoms	Non-cyber victimization	1694	46.65 ± 8.64	6.473	< 0.001
	cyber victimization	160	41.34 ± 10.04		

The Pearson correlation coefficient between Internet addiction and psychological and physical symptoms was calculated (*r* = − 0.374), which identified a negative correlation between Internet addiction and psychological and physical symptoms (*p <* 0.05). These findings indicated that a significant correlation existed between mental and physical health, cyber victimization, and Internet addiction.

### Regression analysis

Multiple linear regression analysis for predicting psychological and physical symptoms indicated that both Internet addiction (β = − 0.278, *p <* 0.001) and cyber victimization (β = − 0.102, *p <* 0.001) significantly and negatively predicted psychological and physical symptoms after demographic variables such as age, gender, being an only child, academic performance, academic stress, physical exercise, and family income were controlled (see Table 3).

**Table 3:** Regression analysis for the prediction of psychological and physical symptoms

Model		Unstandardized Coefficients		Standardized Coefficients	t	*p*-value	Adjusted R^2^
		B	Std. Error	Beta			
1	(Constant)	55.411	0.592		93.614	< 0.001	
	Internet addiction	−0.218	0.014	−0.358	−15.589	< 0.001	0.151
	cyber victimization	−3.426	0.73	−0.108	−4.693	< 0.001	
2	(Constant)	55.411	0.592		93.614	< 0.001	
	Internet addiction	−0.169	0.014	−0.278	−12.5	< 0.001	
	cyber victimization	−3.239	0.689	−0.102	−4.702	< 0.001	
	Age	−0.187	0.12	−0.035	−1.554	0.12	0.106
	Gender	1.729	0.384	0.099	4.505	< 0.001	
	Single child	−0.041	0.358	− 0.002	− 0.114	0.909	
	Academic performance	−0.857	0.164	−0.116	−5.229	< 0.001	
	Academic stress	−2.873	0.341	−0.186	−8.42	< 0.001	
	Physical exercise	1.35	0.226	0.138	5.974	< 0.001	
	Family income	−1.483	0.427	−0.076	−3.473	0.001	

### Mediation model

**Fig. 1:**
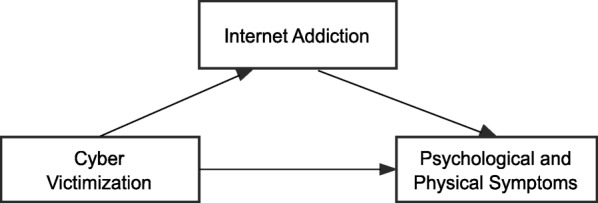
Mediation model in which the independent variable was cyber victimization, the mediating variable was Internet addiction, and the dependent variables were psychological and physical symptoms

The mediating effect of Internet addiction on the relationship between cyber victimization and both psychological and physical symptoms was significant, using of 2000 bootstrap samples, with an indirect effect value and 95% CI = − 1.329 (− 1.904, − 0.820) and the direct effect value and 95% CI = − 2.989 (− 4.283, − 1.696), both 95% CI excludes zero. Thus, the results verified that Internet addiction mediated the relationship between cyber victimization and psychological and physical symptoms (see Fig. 1).

### Moderation model

**Fig. 2:**
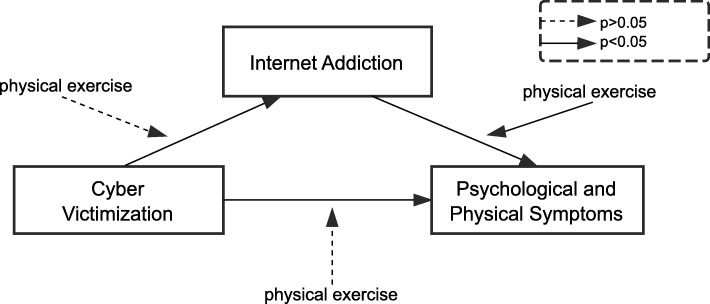
Moderation model in which the moderator was physical exercise and the moderating effect on the three paths was examined. Only one path was significant

The results of the moderation indicated that physical exercise moderated the relationship between Internet addiction and both psychological and physical symptoms (*p* = 0.0474, 95% CI = − 0.484, − 0.0003). However, the moderating effect of physical exercise on the other two paths between cyber victimization and Internet addiction, cyber victimization, and psychological and physical symptoms were not significant (*p >* 0.05) and were not reported (see Fig. 2).

## Discussion

Data from many countries reported the occurrence of cyberbullying victimization at around 15% [2–5], which is considered prevalent. Numerous studies have been conducted to investigate the consequence of cyberbullying, including problems of mental and physical health [3, 4, 6, 15, 47]. Nonetheless, the potential mechanisms underlying cyber victimization and psychological and physical symptoms remain unclear, and only little research has explored this specific topic [13, 21, 22]. The present study is the first to show that internet addiction mediates the relationship between cyber victimization and physical and mental health, and that physical exercise plays a moderating role on the above mediation.

The present study found that victims of cyberbullying had significantly higher rates of Internet addiction than non-cyber victims. This is consistent with previous studies demonstrating that cyber victimization is positively related with Internet addiction [25, 26]. Moreover, this work also suggested that victims of cyberbullying showed lower levels of physical and mental health compared with non-victims. This is in line with many studies that indicated a negative impact of cyber victimization on health [15–17, 19]. The technology advance of the Internet simplifies online victimization by peers, which is accompanied by an increase of suicides because of the suffering caused by cyberbullying [48].

One innovative point of this study is that it successfully associates the mechanism between cyber victimization and both mental and physical health with Internet addiction. This is a new and increasingly prevalent problem among students, and the maximum occurrence of internet addiction has been reported to be almost 47.7% [31]. The newly found mediation in this study suggests that youths who are cyberbullied by their peers could further develop health problems, partly via addiction to the Internet. This implication is based on previous research that demonstrated a positive association between cyber victimization and Internet addiction [25, 26]. This is consistent with the psychological escape hypothesis, which proposes that more time may be spent on Internet gaming to avoid problems that are hard to solve or that create emotional distress [27]. Moreover, Internet addiction has been documented to be significantly related to social isolation [49], physical inactivity [33, 35], body aches, increased body weight [30], and sleep problems [50, 51] which may further contribute to adverse health consequences among the victims of cyberbullying.

In the present study, physical exercise moderated the relationship between Internet addiction and psychological and physical symptoms, which indicates that exercise can alleviate the negative effect of Internet addiction on health. This finding is similar to previous findings that suggested that individuals who addicted to Internet tend to be physically inactive [33–35] and exhibit lower levels of satisfaction from sleep compared with physically active peers [36]. In general, physical activity is assumed to promote physical fitness as a result of the release of neurotransmitters such as endorphins, dopamine, and noradrenaline, induced by exercise, which subsequently promotes mental health [41]. Moreover, a meta-analysis indicated that even patients (e.g. patients with depression) can also benefit from exercise when struggling with psychological symptoms, including depression [52].

Given that the path through which cyber victimization can lead to impairments in psychological and physical health remains unknown, finding further evidence to explain this potential mechanism is central. In the present work, the mediator (i.e., Internet addiction) was proven to be one path with which cyberbullying victims may develop physical and mental health problems. Hence, this path could better explain how youths may cope with cyberbullying and might likewise provide a suggestion regarding behavioral changes so that parents and teachers can perceive and identify signs early. Thus, in clinical practice, addressing Internet addiction among the victims of cyberbullying may break the adverse bidirectional effect and decrease the likelihood of negative mental and physical health outcomes. Furthermore, physical exercise is a convenient and effective way to weaken the negative impacts of Internet addiction on overall health.

Certain limitations affect this work. First, the research is a cross-sectional study; therefore, causation between variables cannot be assumed. Given that the relationship between variables may be bidirectional, the finding that Internet addiction plays a mediating role between cyber victimization and physical and mental health should be considered tentatively. A previous study found that cyber victimization mediates the association between Internet use and mental health problems [53]. Thus, prospective surveys on this topic would be persuasive and necessary in the future [11, 54]. Furthermore, the study design can be further improved by adding the intervening role of physical exercise. Doing so can examine and support the moderating effect of physical exercise on the association between Internet addiction and psychological and physical symptoms, which was found in the present work. Second, cyber victimization was measured over the previous year leading up to the start of this survey. However, psychological and physical symptoms were estimated during the previous month and no exact time period was used to measure Internet addiction. Thus, whether Internet addiction or the associated health outcomes appeared after or before the occurrence of cyber victimization remains unknown. Hence, evaluating the interactions within these variables remains complicated. This limitation could be further promoted by using a detailed questionnaire or a longitudinal design. Third, all questionnaires (including the experience of cyber victimization) were self-rated by the students, which unavoidably leads to a recall bias. Last, in the present study, only a single question was used to assess cyber victimization; however, the adoption of scales that contains several items to measure cyber victimization may improve data quality [6, 26].

## Conclusion

The mediation demonstrated that being subjected to cyberbullying during the teenage years could lead to health problems via Internet addiction. The finding may help health service workers to better understand how victims of cyberbullying deal with this form of bullying, thus developing specifically directed interventions. Furthermore, physical exercise can mitigate the mediating effect of internet addiction on the association between cyber victimization and physical and mental health. Hence, it is advisable to take up physical exercise to help to cope with cyberbullying, while buffering the adverse impact on both physical and mental health.

## Data Availability

The datasets used and/or analysed during the current study are available from the corresponding author on reasonable request.

## Abbreviations

PTSD: Posttraumatic stress disorder
PIU: Problematic Internet use
IAT: The Internet addiction test
WHOQOL-BRIEF: The World Health Organization Quality of Life-BREF
SPSS: Statistic Package for Social Science
95%CI: Confidence Interval
